# Supporting re-engagement with HIV services after treatment interruption in South Africa: a mixed method program evaluation of MSF’s Welcome Service

**DOI:** 10.1038/s41598-024-57774-9

**Published:** 2024-03-27

**Authors:** Kirsten D. Arendse, Caroline Walker, Colin Pfaff, Keitumetse Lebelo, Tali Cassidy, Petros Isaakidis, Erin von der Heyden, Fareed Abdullah, Tom Ellman, Ingrid T. Katz, Jonathan Euvrard, Claire M. Keene

**Affiliations:** 1 Médecins Sans Frontières, Cape Town, South Africa; 2 School of Public Health and Family Medicine, University of Cape Town, Cape Town, South Africa; 3 Clinical and Molecular Epidemiology Unit, Department of Hygiene and Epidemiology, University of Ioannina School of Medicine, Ioannina, Greece; 4 Western Cape Department of Health, Cape Town, South Africa; 5 Office of AIDS and TB Research, South African Medical Research Council, Cape Town, South Africa; 6 Department of Public Health Medicine, School of Public Health and Health Systems, University of Pretoria, Pretoria, South Africa; 7 Division of Infectious Diseases, Steve Biko Academic Hospital and Faculty of Health Sciences, University of Pretoria, Pretoria, South Africa; 8 Department of Medicine, Brigham and Women’s Hospital, Boston, MA USA; 9 Nuffield Department of Medicine Centre for Global Health Research, Health Systems Collaborative, University of Oxford, Oxford, UK

**Keywords:** Medical research, Translational research

## Abstract

Psychosocial challenges impact patients’ ability to remain on antiretroviral therapy lifelong, magnified by disorganized health-systems and healthcare worker (HCW) attitudes. To address this, Médecins Sans Frontières and the Department of Health developed the *Welcome Service* intervention, to provide person-centered care at re-engagement after HIV treatment interruption. Implemented in Khayelitsha, South Africa, between August 2020 and February 2021, the intervention aimed to reorganize triage, optimize clinical and counselling services and address HCW attitudes. The study used a mixed-methods design, incorporating in-depth interviews, and analyses of programmatic and routine health data. Interviews demonstrated positive patient care experiences. HCWs understood the potential impact of attitudes on patient engagement, however, some continued to demonstrate judgmental attitude. Clinical objectives were variably met at re-engagement: 98% were re-initiated the same day, 50% had a CD4 done, and 45% received tuberculosis prevention. Nevertheless, 4-month retention was 66%, and 88% had a VL < 1000 c/mL. Despite HCWs’ understanding of person-centered care not translating into supportive behaviors, patients had positive care experiences and the intervention ended with a high rate of VL suppression. More efforts are needed to design interventions building on *Welcome Service* principles to provide person-centered care and sustain retention after re-engagement.

## Introduction

Access to antiretroviral therapy (ART) in South Africa has dramatically improved over the past 20 years, however, progress toward the Joint United Nations Program against HIV/AIDS 95–95–95 targets is diminished by the ongoing challenges associated with treatment interruption^1^. In South Africa in 2021, an estimated 7.2 million people were living with HIV: 95% knew their status, of which 77% were on treatment and 92% of those were virally suppressed^1^. These targets assume that people experience HIV in a linear and unidirectional manner, where they are diagnosed with HIV, start treatment, and become virally suppressed^2^. In reality, people cycle in and out of care throughout their treatment journey when competing psychosocial, environmental, and economic challenges impact their ability to prioritize health^2–7^. While HIV has transitioned from a life-threatening, short-lived illness to a chronic disease with potentially near-normal life expectancy^4–7^, literature shows that disengagement from HIV care is common, with 25% of people on ART in South Africa having a treatment interruption of 6 months or more during a 2-year period^5^.

ART interruption is associated with an increased risk of opportunistic infections, mortality, and transmission^4,6–8^. Additionally, disengagement can lead to treatment resistance, necessitating switches to less tolerable ART regimens with higher pill burden^4,8,9^. People who disengage can be time- and resource-intensive to manage, as they may require tracing and re-linkage to care, more intensive medical care for advanced HIV, and costly second or third-line regimens^5^. This increases the burden on already overstretched health systems, particularly in resource-limited settings. A study in South Africa showed that 32% of people with HIV admitted to an emergency unit had interrupted treatment, both demonstrating the high burden of ART interruption and advanced HIV on health systems^10^. ART interruption may have been further impacted by the COVID-19 pandemic, where public health resources were redirected to the emergency response^11^.

In many contexts including South Africa, the HIV burden disproportionately affects people living in poverty facing challenging social circumstances^12^. Commonly, people experience competing psychosocial and socioeconomic challenges that affect their ability to remain engaged in HIV care^3,13^. This is further complicated by inefficient clinic systems, long waiting times, far travelling distances to access care, and stigmatizing healthcare workers’ (HCWs) attitudes, particularly towards those who have interrupted treatment^3,13,14^. Previous research shows HCWs, who function as gatekeepers and determine who receives care, can inadvertently provide care that proves to be stigmatizing, or viewed as “punishing”^3,15^. These negative attitudes and behaviors, in addition to uninviting, inefficient, and complex clinic systems, can deter patients from accessing treatment, further driving disengagement^3,15–17^. Few interventions have attempted to target friendliness and efficiency of HIV health services as strategies to improve the experience of re-engagement and promote long-term retention in care.

Although many strategies have been adopted to improve ART uptake globally, such as reduced pill burden, improved drug tolerability, widespread ART rollout through ‘Universal Test-and-Treat’, and differentiated service delivery models, many unresolved systemic challenges make sustained long-term engagement difficult^18,19^. Health systems are not designed to adequately support patients when they re-engage after an interruption, particularly as they are more likely to suffer from complicated psychosocial barriers that led them to disengage, many of which may be out of the health system’s control^3^.

Recognizing the need to tackle disengagement, Médecins Sans Frontières (MSF) and the Western Cape Department of Health (DOH) designed and implemented the *Welcome Service* intervention to support people re-engaging with primary care services after ART interruption, aiming to improve treatment outcomes. This study is a programmatic evaluation of the *Welcome Service* intervention.

## Methods

### Study design and setting

The primary aim of this study was to evaluate implementation of the *Welcome Service* intervention. This was done using a convergent, mixed methods design, incorporating an analysis of in-depth qualitative interviews with HCWs and re-engaging patients enrolled into the program and a descriptive quantitative analysis to describe the process of implementation and the clinical outcomes of those enrolled in the *Welcome Service*. The secondary aim of the study was to conduct a pre and post analysis of clinical outcomes using aggregated clinic-level routine health data, among all people accessing HIV healthcare services at the intervention sites, irrespective of whether they had an interruption in care or received the intervention.

The intervention was implemented at two primary healthcare clinics (intervention clinics) in Khayelitsha, a peri-urban informal settlement outside Cape Town, South Africa, with over 450,000 residents. Community members face challenges such as: limited access to piped water and sewerage, living in informal dwellings, and high rates of unemployment (up to 34%)^20^. HIV prevalence is 13.7% among the South African adult population, with Khayelitsha’s prevalence above the national average^21,22^. For the pre-post analysis, we compared clinic-level ART outcomes at the intervention sites with those of two clinics in Cape Town where the standard of care services was received. These clinics were chosen based on similar demographics, unemployment rate, socioeconomic status, living conditions, and HIV prevalence^23^.

### Intervention development and implementation

Development of the intervention’s key principles as well as its design involved consultation with a patient advisory group and stakeholders (including the DOH, local clinic staff, and other not-for-profit organizations). This included implementation of a pilot project, and review and feedback of monitoring and evaluation data with stakeholders before implementation at the study sites. Implementation at the study sites took place between August 2020 and February 2021. While the intervention was run by clinic staff, the implementation team continued data collection and provided strategic support until project closure on 30 June 2022. Adaptations were made based on resource availability, stakeholder buy-in, experience of clinic staff and the implementing team, contextual changes, and shifts in healthcare service provision due to COVID-19. The *Welcome Service* intervention’s design was premised on addressing factors shown to impact ART engagement that are within the health system’s control to address^3^. Five key principles were developed: (1) WELCOME: people should be welcomed by HCWs at re-engagement and made to feel valued, (2) NORMALIZE: struggling with engagement should be normalized to reduce guilt and stigma around disengagement, (3) ACKNOWLEDGE: a person’s decision to return to care should be celebrated and a treatment plan should build on this, (4) SUPPORT: provide psychosocial support to manage challenges to remaining engaged in care, and (5) EMPOWER: help people to take ownership of their treatment (Supplement 1.1).

The service had four core implementation objectives. These were approached using a variety of implementation strategies as described in Table 1, drawing on Proctor and Leeman’s frameworks^24,25^. Clinical and counselling packages were designed in consultation with stakeholders and the People’s Development Centre (PDC), following local HIV guidelines. The PDC is a branch of the Western Cape DOH that conduct training for nurses and counsellors in the province. After the intervention, the training packages were incorporated into PDC’s nurse and counsellor training curricula and can be made available on request. The core principles, emphasized in training sessions, are described further in Supplement 1.1 and 2 and in the open access training materials^26^. Nurses received training and mentorship to enhance their skills in assessing and managing disengagement, aligned with local HIV guidelines. Adherence counsellors underwent training and mentorship to address client-specific barriers to engagement. Training was provided to all clinic staff, including clinical and support personnel on how to deliver care that is person-centered. This workshop aimed to build a deeper understanding of patient values and address negative attitudes and behaviors portrayed towards people who disengage and encourage the embodiment of the 5 core principles in everyday patient-provider interactions. It also aimed to change the language HCWs used, such as referring to ‘disengagement’, moving away from the judgmental ‘defaulting’.

**Table 1:** Overview of implementation objectives, strategies, actors, targets, and measures used to evaluate implementation and clinical outcomes.

Implementation objectives and proposed mechanisms to improve clinical outcomes	Implementation strategies	Actors and targets	Measures to evaluate implementation	Measures used to evaluate outcomes
(1) Improve the identification, triage and monitoring of re-engaging patients Reduce delays and streamline services Prevent disengaged patients from being seen last or turned away Prioritize ill patients through triage Improve monitoring and evaluation of disengaged patients with electronic records	Restructuring: Mapping patient flow and clinic reorganization to ensure the direction of patients into the appropriate services Capacity building: Training data clerks, triage, and admin staff to identify patient on re-engagement, ensure everyone is treated the same day, acute cases are prioritized and ensure capturing takes place for monitoring and evaluation	*Actors* Program manager, Epidemiologist, data/clerk mentors *Targets* Data clerks, triage staff	Qualitative: Describe HCWs' and patients’ experiences of the *Welcome Service* using in-depth interviews, focusing on mapping, flow, reorganization, monitoring and efficiency Quantitative: (i) Number enrolled in the service (ii) Number eligible for enrolment (had an interruption in care ≥ 56 days at the intervention clinics)	Not evaluated
(2) Optimize the clinical management of re-engaging patients by improving fidelity with provincial HIV guidelines^28^, specifically: Re-initiate ART early and safely after interruption Switch regimen early and appropriately if needed Screen for and prevent opportunistic infections	Information dissemination: Workshops for doctors and nurses managing patients working with PLHIV Capacity building: One-on-one mentorship for nurses working with PLHIV, with a focus on managing disengaged patients	*Actors* Nurse mentors, program Manager *Targets* Nurses and doctors	Qualitative: Describe HCWs’ experiences of managing disengaged patients using IDIs, focusing on clinical care and endorsement of recommendations Quantitative measures at/after re-engagement (the enrolment visit): (i) Time to ART restart (Proportion restarted on ART the same day as re-engagement) (ii) Had a CD4 count at re-engagement (iii) Received cotrimoxazole, if applicable (iv) Received TB prevention therapy, if applicable (v) Had a VL at 4 months after enrolment (vi) Regimen and re-initiation (Proportion restarted on first-line with dolutegravir)	Outcomes mong patients in the *Welcome Service:* 1. Retention in care 2. VL Outcomes at clinic-level among all ART patients at the intervention sites and comparison clinics: 1. Retention 2. Medication possession ratio 3. VL
(3) Improve psychosocial support for re-engaging patients Improve the identification and management of patient-specific barriers to engagement Refer patients to mental health and social services where indicated (social work, substance use, gender-based violence etc.)	Information dissemination: Workshop for counsellors on managing disengagement Capacity building: One-on-one mentorship with a counsellor educator using a stationary tool developed for the intervention	*Actors* Counsellor mentors *Targets* Adherence counsellors	Qualitative: Describe HCWs’ experience of managing patients who re-engage, and patients’ experience of the counselling, coping mechanisms, referrals and advice received from HCWs through in-depth interviews	Not evaluated
(4) Address negative attitudes and behaviors HCWs may portray towards re-engaging patients Engage HCWs’ stressors and provide tools to cope Acknowledge and support HCWs’ challenges with disengagement Recognize how stress and burnout can affect patient care Promote a more welcoming environment and non-judgmental attitude toward re-engaging patients	Information dissemination: A one-day workshop designed focused on empathy training and values clarification, using behavior change techniques In objectives 1–3, consideration of attitudes and behaviors was incorporated in the information and support given throughout training, mentorship and support and emphasized in the day-to-day operations	*Actors* Workshop facilitators *Targets* All HCWs (clinical and support staff)	Qualitative: Describe HCWs’ perception of disengagement and their experience of managing re-engaging patients and patients’ experiences of engagement with interviews	Not evaluated

*ART* Antiretroviral Therapy, *HCW* Healthcare Worker, *IDI* in-depth interview, *VL*  viral load.

### The intervention

**Figure 1:**
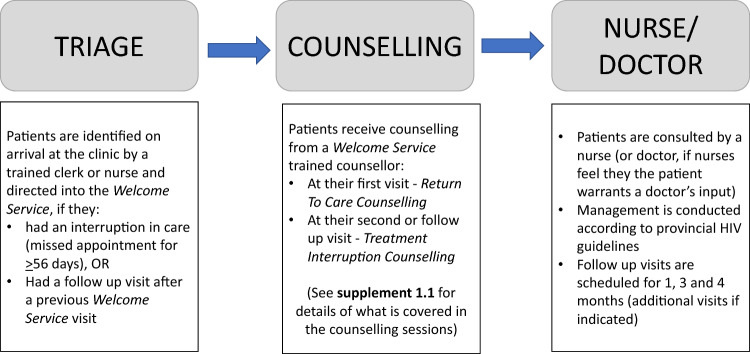
Overview of patient flow in the welcome service.

Patients re-engaging with care at the intervention clinics after a period of ART interruption for ≥ 56 days were enrolled in the *Welcome Service*, where they would receive care at scheduled visits for at least 4 months before going back into routine health care services. In routine health services in South Africa, medication is dispensed as a pack of 4 weeks (28 days); follow up visits are scheduled as multiples of 28 days (1 month = 28 days, 2 months = 56 days etc.) to align with pharmacy collection days. In the Western Cape, the maximum packs of ART that can be dispensed at one time is 2 × 28 days. Figure 1 demonstrates the three steps a patient would encounter at each *Welcome Service* visit (including subsequent follow-up visits). (1) Triage: patients arrive at the clinic and are identified by a trained nurse or clerk through triage and enrolled into the *Welcome Service* (or at follow-up, they are identified as having a follow-up visit). (2) Patients receive counselling by a trained counsellor—two types of sessions are provided (visit 1: return to care counselling and visit 2: treatment interruption counselling, described further in Supplement 1.1). (3) Patients receive guideline-supported clinical care by a trained nurse, who would aim to address the clinical objectives outlined in Table 1 (including same-day restart, CD4 testing, switch to dolutegravir if indicated, etc.). Clinical management was the same as routine care as per guidelines, however, the intervention provided additional clinical and training tools for nurses to support their implementation. Follow-up visits were usually scheduled at 1, 3 and 4 months after re-engagement, unless additional visits were clinically indicated, such as for those who were not re-started on ART at the enrolment visit or required more regular follow up for an opportunistic infection. The intervention was integrated into routine care and was aimed at all patients re-engaging with care at the clinic.

### Data sources and analyses

**Figure 2:**
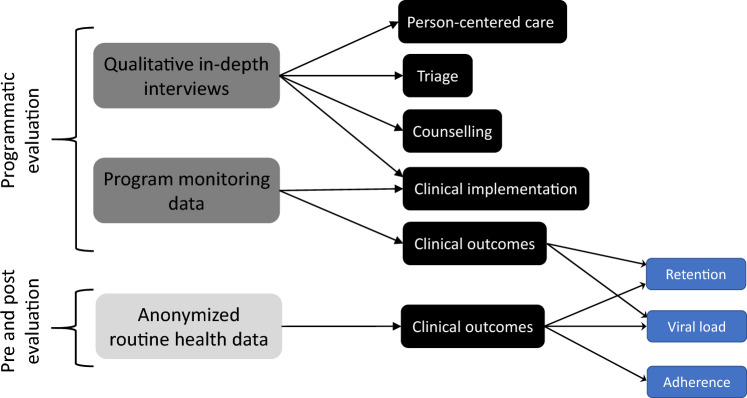
Overview of data sources used for evaluation.

For the primary analysis (i), we conducted a descriptive evaluation of implementation, using two data sources: qualitative in-depth interviews and collected quantitative program-monitoring data. For the secondary analysis (ii), we conducted a pre- and post-evaluation of clinic-level ART outcomes using anonymized, routinely collected health data (See Fig. 2 and Supplement 1.2 for descriptions of the data sources). Owing to the anonymity of the routine health data, it could not be linked to the data used in the primary analysis.

#### Primary analysis: descriptive mixed-methods evaluation

*Data source 1—qualitative data*: Using purposive sampling, we conducted once-off individual interviews with HCWs at the intervention clinics who were trained on the *Welcome Service* and re-engaging patients who received the intervention, using topic guides until saturation was reached, exploring participants’ experiences of the intervention. During HCW interviews we explored their knowledge and understanding of the intervention principles, including their understanding and value of the various elements implemented, with a strong focus on exploring attitudes and behaviors toward patient disengagement from care, thereby aiming to evaluate the specific teachings in the person-centered care training. Design of topic guides was influenced by an evaluation of HCWs’ perceptions of disengagement amongst the same population conducted before the intervention^15^. Topic guides were pretested, using separate guides for HCWs and patients, and revisions were made after debriefing from pilot interviews. HCWs and patient topic guides had some overlapping questions on knowledge of the intervention’s content and their experience within it. Patients were specifically asked about their HIV diagnosis and care experiences, while HCWs were asked about their roles and specific challenges associated it. Interviews were conducted between December 2021 and May 2022 by NZ, a member of the study team, in isiXhosa or English (based on participant preference), audio-recorded, translated, and transcribed. Participants were recruited by a nurse in the study team who would take their contact details after receiving care if interested and were contacted by NZ to arrange an in-person interview. No compensation was provided to HCWs, and patients were reimbursed for transport to attend interviews. Data was collected and analyzed in line with the consolidated criteria for reporting qualitative studied^27^.

*Data source 2—quantitative program monitoring data*: We collected quantitative data from patients enrolled into the *Welcome Service* between 01 April and 30 June 2021 (the study enrollment period), until 6 months after the enrolment date for each participant. Counsellors kept a daily record of which patients enrolled into the *Welcome Service*. Data capturers used this list and manually searched records using the clinic’s onsite digitized system, the primary health care information system (PHCIS) to gather data on patients’ clinic visits, pharmacy, and laboratory records. Data was captured and stored in REDCap. Data was collected to provide insights into the implementation process and describe clinical outcomes, including clinical objectives (e.g. time to ART re-initiation, ART regimen at re-initiation, visits attended, CPT/TPT prevention) and clinical outcomes (retention and VL suppression) based on recommendations in the Provincial HIV guidelines (Table 1)^28^. Data were collected and reviewed quarterly, and findings presented to the MSF team, Western Cape DOH, and clinic staff.

*Primary data analysis*: Manual coding was done using Microsoft Word templates developed for the study by CW, supported by NZ, and discrepancies discussed between them. Thematic analysis was used to categorize key elements of HCWs’ and patients’ experiences of the intervention^29^. Transcripts were used to ascertain emerging themes and co-create a codebook. Codes and key themes were compared and discussed, and a summary written, shared, and discussed with co-authors before writing. Descriptive statistics and qualitative findings were triangulated following a convergent mixed methods approach, allowing for consideration of quantitative and qualitative findings to evaluate the intervention, with elements of each dataset complementing the other^30^. Theoretical assumptions and execution of all elements of the intervention were considered and analyzed. Data sets were summarized, presented, and discussed among co-authors before writing, with subsequent revisions and discussions helping to finalize the analysis based on group consensus. When concerns or gaps were highlighted, data sources were revisited, including the revision of transcripts, coding frameworks, and quantitative data analyses^31^. Quantitative data was managed and analyzed using Stata 17.0^32^.

#### Secondary analysis: pre and post evaluation of clinic-level ART outcomes

*Data source*: Anonymized, routinely collected health data were provided by the Provincial Health Data Center (PHDC). The PHDC is a routine health-data repository managed by the Western Cape DOH that consolidates health information from a variety of existing routine data systems, and links data at patient level^33^.

**Figure 3:**
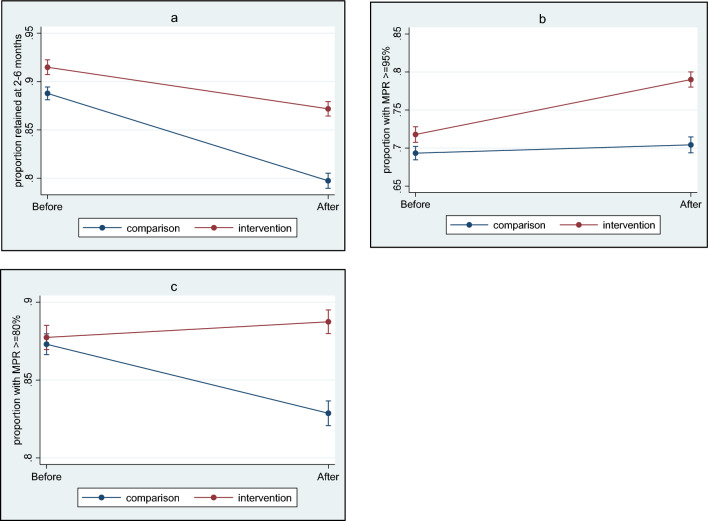
(**a**–**c**) Demonstrating clinic-level outcomes (retention and medication possession ratio) among all patients accessing antiretroviral services at the intervention and comparison clinics, before and after the implementation: (**a**) Proportion with 4-month retention at the intervention and comparison clinics before and after implementation. (**b**) Proportion with a 0–6-month medication possession ratio of ≥ 95% at the intervention and comparison clinics before and after implementation. (**c**) Proportion with a 0–6-month medication possession ratio of ≥ 80% at the intervention and comparison clinics before and after implementation.

*Quantitative data analysis*: We compared clinic-level ART outcomes before and after the intervention, reporting on 4-month retention, adherence, and VL outcomes (See Box 1). To observe trends over time and account for baseline clinical outcomes, we measured ART outcomes at both the intervention clinics and two non-intervention clinics, using a difference in differences (DID) analysis. Patients were included in the analysis if they had an HIV-related healthcare visit (i.e. an HIV-related blood test or an ART regimen dispensed) during the study enrolment period at the intervention or non-intervention clinics and were ≥ 18 years, irrespective of having an ART interruption or receiving the intervention. All multivariate analyses accounted for sex, age, and ART regimen. Statistical significance was considered at 5%. For each outcome variable evaluated, parallel trends were tested, and, if met, statistics reported. Data cleaning, analyses, and graphs (Fig. 3a–c) were conducted using Stata 17.0^32^.

**Box 1:** Key definitions.

**Retention:** this was defined as any HIV-related healthcare visit (excluding hospital admissions) at 4 months after the first visit during the study period, with a window of 2 months before and after. This period was selected because the intervention program was designed to provide support for approximately 4 months per individual **Adherence/Medication possession ratio (MPR):** this was measured between 0 days and 6 months (180 days, the upper limit of the retention window) following each participant’s first visit during the study, calculated as the number of days of ART dispensed divided by the number of days during that period between visits for that individual (Supplement 1.3). Although MPR does not measure whether patients took the treatment (*secondary adherence*), it provides an estimate of the best possible adherence, *primary adherence*^55^. We report *adherence* as the proportion of people who had an MPR ≥ 80% to reflect newer literature as well as the historically used threshold of ≥ 95%^60^ **Viral load (VL) suppression:** because local HIV guidelines state that VL should be conducted 3–4 months after re-initiation of ART, we evaluated the most recent VL at this time point after *enrolment*, with a window of 2 months before and after^28^. VL *suppression* is reported at two thresholds: < 50 copies/mL and < 1000 copies/mL, to increase comparability to other literature as both are widely used	

### Ethics considerations and consent

This study was approved by the Human Research Ethics Committees of the University of Cape Town (Protocol Number: 542/2019) and Médecins Sans Frontières Ethics Review Board (Reference: 1947). Patients receiving the *Welcome Service* intervention were not required to provide consent for participation because the program was implemented as part of routine care at the intervention clinics, in collaboration with the DOH. The data were collected as part of program monitoring and evaluation, and quality improvement, therefore individual consent was not required. Written informed consent was obtained from all interview participants in English or isiXhosa based on their preference. Patient consent for access to the anonymized, routine health data was not required, in line with Sections. 15 and 16 of the South African National Health Act.

## Results

### Primary analysis: programmatic evaluation of implementation

521 people were enrolled in the *Welcome Service* between 1 April 2021 to 3 June 2021; 68% were female and median age was 38 years (interquartile range, IQR, 32-44) (Table 2). Of 30 participants interviewed, 18 were re-engaging patients, and 12 were HCWs (4 nurses, 6 counsellors, and 2 data clerks). The headings below are key elements of the intervention as well as major themes which arose from interviews.

**Table 2:** Characteristics and clinical outcomes of re-engaging patients enrolled into the *Welcome Service* using program-monitoring data.

Variable			Number (%)	
Total patients, n			521	
Female, n (%)			355 (68)	
Age in years, median (IQR)			38 (32–44)	
Restarted on ART the same day, n (%)			513 (98)	
Number eligible for repeat CD4 at re-engagement, n (%)			466/521 (89)	
CD4 within 2 months of re-engagement if eligible, n (%)			257/466 (50)	
CD4 count (cells/mm3), n (%)	< 50		16 (6)	
	50–100		16 (6)	
	100–200		41 (16)	
	200–350		55 (21)	
	> 350		129 (50)	
Received cotrimoxazole prevention at re-engagement, n (%)			233/521 (45)	
ART regimen, n (%)			Before interruption	At re-engagement
	First-line with NNRTI		303 (58)	230 (44)
	First-line with dolutegravir		138 (26)	210 (40)
	Second-line with protease inhibitor		44 (8)	43 (8)
	Second-line with dolutegravir		11 (2)	11 (2)
	All other regimens		25 (5)	27 (5)
Eligible for TPT, n (%)			259 (50)	
Received TPT after re-engagement if eligible, n (%)			113/259 (44)	
Visit attendance between 2 and 6 months after re-engagement			342 (66)	
VL complete between 2 and 6 months after re-engagement (all patients)			210/521 (40)	
VL complete amongst those retained in care (between 2 and 6 months after re-engagement)			210/342 (58)	
VL distribution in copies/mL	< 50		85 (40)	
	50–999		100 (48)	
	> 1000		25 (12)	

*ART*  antiretroviral therapy; *IQR* Interquartile Range, *NNRTI * Non-Nucleoside Reverse Transcriptase Inhibitor, *n * Number, *TPT* Tuberculosis prevention therapy; *VL* = viral load.

#### Awareness of the intervention

Understanding of the intervention was inconsistent across participants. All counsellors were familiar with the program’s aims and components, whereas nurses presented less complete descriptions. About half of the patients were aware of what the intervention was, what it offered, or that it was different from standard of care. Most of these participants only discussed the intervention when prompted with specific questions about the *Welcome Service* by the interviewer, despite an explicit mention of it in the introduction of the interview. Participants were then positive or neutral in their response.

“It [the Welcome Service] was organized for people that had defaulted and came back. That they don't feel discriminated against for skipping their treatment. And to check on their experiences about when they came back after skipping their treatment. [sic] That they must still feel welcomed, and not made to feel as though they did something wrong at the time.” (Female patient, 34 years)

#### Identification, triage, and flow

Re-engaging patients described experiences before the intervention of long waiting times and indirect punishment by being seen last if they missed an appointment, citing this protracted time in the clinic as a barrier to re-engagement. However, both HCWs and patients described smoother clinic flow, better file management and decreased waiting times.

“We try to make things easier for patients” (Female data clerk, 47 years)

“Going from the counsellors straight to us [nurses]” (Female nurse, 55 years)

“If they come now and their folders are there, there's no such thing as “let me see when last this person visited the clinic”, no. They will be called in nicely, just like everybody.” (Female counsellor, 34 years)

#### Clinical management and fidelity to provincial HIV guidelines

Most nurses felt their role in the *Welcome Service* was no different to routine care. No mention was made of any changes in interaction with patients. Any description of non-clinical work done by nurses was presented as standard of care rather than *Welcome Service*. Rather, they perceived the *Welcome Service* as counselling-focused and thus an intervention more relevant for the counsellors.

“We have been doing this Welcome Service. It’s just that we didn’t have no name before.” (Female nurse, 56 years)

“They take bloods, go for counselling, get counselling, and get a chance to talk about their problems. From there they go to the Sister and the Sister will fill in the gaps left from counselling.” (Female nurse, 61 years)

Of the 521 patients were manually captured in the program monitoring data, 98% restarted ART the same day they re-engaged. Although most nurses did not see the *Welcome Service* as something new, one highlighted that training helped her to manage people with advanced HIV, while another praised same-day ART restart as an emphasized change in the *Welcome* Service that allowed for reduced visits*,* whereas previously they would have come back for results before re-initiation, despite guidelines recommending rapid restart. Patients did not perceive any change in clinical management beyond their usual expectations.

“The Welcome Service has made a difference. At least now a person that has not been here in a long time would be seen by a nurse, a counsellor and then receives treatment on the same day. That same-day treatment...you see before, we would just take their bloods and they would come back in three days’ time.” (Female nurse, 56 years)

Other clinical measures used to evaluate implementation were partially complete: only half (257/466, 50%) of the *Welcome Service* patients eligible for a repeat CD4 count had the test within 2 months of re-engagement. Of those with a CD4, 29% (73/257) had a CD4 < 200c/mm^3^. In addition, only 45% of all patients (233/521) received cotrimoxazole prevention, despite guidelines recommending that all patients receive it at re-engagement, irrespective of previous CD4 counts^28^. Half (45%, 259/521) of re-engaging patients were eligible for tuberculosis (TB) prevention, while only 44% of those (113/259) received it. The proportion of patients restarted on a first-line regimen with dolutegravir increased after the intervention (from 26 to 40%). Of the 521 re-engaging patients, 66% were retained at 4 months after re-engagement, and of those, 58% had a repeat viral load (VL) during the follow up period. Of those with a VL, 40% were undetectable (≤ 50 c\/mL). Using the 2023 WHO definition of VL suppression of < 1000 copies/mL^34^, 88% were suppressed (Table 2).

#### Counselling

“[The Welcome Service] gave me the chance to breathe.” (Female patient, 34 years)

HCWs articulated counselling as the core component of the *Welcome Service*. Both HCWs and patients appreciated its value, yet no clear change in the method or content was observed by patients. Despite introducing interviews as research, some patients saw the sessions as counselling and sought clinical or psychological advice. Counsellors valued the *Welcome Service* as an opportunity to “*sit down with somebody… and just talk” (Female counsellor, 50 years)*. Compared to other participants, counsellors more commonly mentioned the *Welcome Service* unprompted and in positive terms: *“All clinics need the Welcome Service.” (Female counsellor, 42 years).* One counsellor reflected on the positive impact of training on patient interactions. She articulated a change in the counselling approach from close-ended questions and interrogation of patients’ validity for disengagement to creating a safe space for patients to discuss their challenges.

“Before the MSF training [sic] we didn't do things this way... The Welcome Service, I'd say it's a gift that's perfect for dealing with patients that have disengaged. Before it was questions like “You are this person, you last came to the clinic when, you were supposed to come back when but didn't come back, why didn't you come?” You write just those things down. You don't teach them anything.” (Female counsellor, 47 years)

#### Healthcare worker attitudes and behaviors toward disengagement

A key teaching in the *Welcome Service* training and workshops was normalizing the difficulty patients have with taking life-long ART, emphasized using the phrase *“It Is Normal to Struggle”*, although, no patients receiving the service had heard the slogan. One counsellor shared that the *Welcome Service* “*made us to understand that “It Is Normal to Struggle with the Treatment”” (Female counsellor, 34 years)*. However, most counsellors and nurses stated they did not agree with this sentiment. They inferred a lack of trust in patients and saw the slogan as condoning disengagement. Disagreement with the statement was fueled by concerns of giving patients ‘permission’ to make poor health choices, meaning the statement should not be communicated.

“Interviewer: What do you think will happen if you tell a patient that it is normal to struggle with taking treatment?

Participant: It will make the patient feel good because “you haven't done anything wrong”. [sic] as much as you say that [it is normal to struggle] you say it with a pinch of salt because you don't want the patient to think “It's fine, I can default, I can disengage, they will understand”. It's not about who’s understanding, it's about what's going on in your body as a patient.” (Female nurse, 50 years)

Central to the interviews was this concept of struggling and validating the struggle. HCWs had differing views on what ‘struggling’ meant, ranging from: disengaging from care, difficulty taking pills or having an unsuppressed VL. HCWs often expected patients to give reasons for disengagement and, commonly, passed a degree of judgment as they would legitimize some reasons and invalidate others. Counsellors appeared more generous than other HCWs in what they believed were valid reasons for disengagement.

“I get very concerned when someone disengages…. Some have vague reasons. A person would say they have nothing to eat. The situation at home...I'd [sic] would think to myself that if I was in that situation, I wouldn't find this to be a problem. There is no one that gives a ‘valid, valid reason’. You find that it's weak reasons because when you talk to them versus the problem they are stating, it's not something that would cause them not to take treatment.” (Female nurse, 56 years)

HCW attitudes, strongly emphasized in the training, arose commonly in interviews. Patients’ present tense descriptions made it difficult to ascertain whether this was a concern of the past or continued after implementation*.* Both patients and HCWs referred to HCWs shouting, insulting, and patronizing patients, *“[treating us] like a child” (Female patient, age unknown),* while some HCWs blamed patients for their situation.

“It's the patients themselves [to be blamed]. We are not to be blamed. Because we are here every day.” (Female nurse, 55 years)

Patients shared their experiences of this, commonly focusing on fears of poor treatment, *“expecting…to be shouted at” (Female patients, 36 years)*, and acknowledging these as strong barriers to reengagement. Nevertheless, no instances were reported in recent care experiences, and contrary to what they expected, patients highlighted positive engagement with HCWs.

“Instead of asking me why I hadn't come for such a long, she just showed her joy at the fact that I had returned” (Female patient, age unknown)

**“**But sometimes there are words that you feel are not alright. We are not treated good, even if you defaulted for some days, you become scared of going back to the clinic because you know you will be shouted at, insulted and all that. So those are some of the things that make us to default and not take our treatment the proper way. But I like it now since I've joined the Welcome to Service because we are treated well.” (Female patient, 31 years)

Nurses highlighted barriers limiting in-depth interactions with patients. They felt time-pressured by systemic factors, including human resources constraints and pressure to reach daily consultation targets.

“Because what we are doing in our days is only about chasing the numbers. Which is sometimes you can miss a lot of things by chasing numbers.” (Female nurse, 55 years)

“There's too many patients and too much panic…We are short staffed, I won't lie” (Female nurse, 61 years)

Another key learning piece from the *Welcome Service* advocated for a change in terminology, from using the derogatory term ‘defaulting’ to ‘disengaging’. Challenges arose in participants’ understanding of this, particularly in translation to and from English. One counsellor highlighted the change in approach to patients accompanying the change in jargon.

“...even with HIV what makes other people to default, I mean not taking their treatment as they should. We were told not to say they defaulted but disengaged on their treatment, laughter...” (Female counsellor, 50 years)

### Secondary analysis: pre and post intervention evaluation of clinic-level outcomes

Clinical outcomes (retention, adherence and VL suppression) among all people accessing ART services at the intervention and comparison clinics are compared using a DID analysis, including all patients irrespective of having an ART interruption or enrolling in the *Welcome Service*. Of the 7623 patients accessing ART services at the intervention clinics during the study period, majority were female (73%), and median age was 42 years (IQR: 36–48). Similar demographics were found among those accessing ART at comparison clinics (Supplement 1.4).

*Retention in care*: Clinic-level retention at 4 months was 87% at the intervention and 80% at the comparison clinics. Both showed a decline in retention compared to baseline before the intervention (Fig. 3a), although this decline was slower at intervention clinics (*p* < 0.001).

*Adherence*: Medication possession ratio (MPR) was used as an approximate measure of *adherence*, defined as the ratio of the amount of medication a patient has in their possession to the number days over a defined period (Box 1). The intervention and comparison clinics showed an increase in 6-month *adherence* after the intervention when using the MPR threshold of ≥ 95% (Fig. 3b). However, this increase was greater at the intervention clinics (7% increase, from 72 to 79%) compared to comparison clinics (1% increase, from 69 to 70%) (*p* < 0.001). Using an MPR threshold of ≥ 80%, an increase was also seen among the intervention clinics compared to baseline (1% increase, from 88 to 89%), whereas a decline was observed among the comparison clinics (4% decline, from 87 to 83%) (Fig. 3c), suggesting an association between the intervention and improved MPR “adherence” at clinic-level (*p* < 0.001) (Supplement 1.4).

*VL suppression*: Half or fewer patients (49–52% at the intervention, 40–41% at the comparison clinics) had a VL conducted between 2 and 6 months after their first visit during the study period, although the proportion of patients who had an indication for VL during this period was unknown. Among those with a VL available, an increase in suppression (< 50c/mL) was seen after the study period among both the intervention (1% increase, from 84 to 85%), and comparison clinics (3% increase, from 84 to 87%). This was similarly seen at the VL suppression threshold of < 1000 c/mL (intervention clinics increased from 93.8 to 95.5% and the comparison clinics increased from 93.2 to 93.9%).

*Parallel trends*: Parallel trend assumptions were met when assessing adherence using MPR thresholds ≥ 95% and MPR ≥ 80% and for 4-month retention. Assumptions for VL suppression were not met, therefore comparisons could not be made using the DID analysis (Supplement 1.5).

## Discussion

This study evaluated implementation of the *Welcome Service* and describes clinical outcomes of those enrolled using a mixed methods analysis. Findings showed variable achievement of implementation objectives, from partial knowledge of the intervention among HCWs and positive patient care experiences, to moderate achievement of clinical objectives and ongoing judgmental HCW attitudes. Nevertheless, 66% of enrolled patients were retained at 4 months, 58% had a repeat VL at 4 months, and of those, 88% had a VL < 1000 c/mL.

Friendly healthcare can positively influence health outcomes, holds significant value in social relations and is well-recognized as a priority for HIV service delivery^35–38^. Patients overwhelmingly emphasized historical experiences of anticipated poor treatment, long waiting times, and inefficient clinic systems as barriers to care. Contrary to reported expectations, patients had positive care experiences with no recent reports of judgmental treatment after the intervention. Anticipated negative treatment and the ‘punishing health system’ can delay care seeking, even in the absence of first-hand experience of poor care^3,39–41^. Anticipated stigma should be addressed by improving quality of care and HCW friendliness, as well as targeted campaigns to manage public fears of HCWs attitudes^42^.

HCWs showed an understanding of patient realities and the potential impact of negative, judgmental HCW attitudes on patient engagement with HIV care, although none admitted to enacting negative behaviors themselves, rather, some showed ongoing judgment by demanding justifiable reasons for disengagement. This suggests a misalignment between understanding the intervention’s core principles and HCWs’ reflections on their own behavior, particularly among nurses who saw their role as ‘no different to routine services’. Addressing attitudes and behaviors may require more in-depth work and empathy training; incorporating these principles into medical curricula could be a valuable step forward^43^. A study exploring HCW attitudes among this population before the intervention showed HCWs had contradictory feelings of empathy and anger towards disengagement: HCWs felt frustrated about disorganized systems and high patient demand, which could be misdirected toward re-engaging patients, who may have more demanding clinical and psychosocial needs^15^. Despite system pressures potentially influencing full adoption of welcoming attitudes, specific elements of the program (perceived decreased waiting times, increased same-day ART, appreciation of counselling) were positively noted by HCWs. Future models to support re-engagement should recognize and tackle root causes of HCWs’ frustrations, such as improving the circumstances in which HCWs practice, address societally shaped stigma and bridge the gap between HCWs’ understanding of patient challenges and empathetic behavior. The “Person-centered care training” offered to all clinic staff, focused on empathy, and instilling a deeper understanding of patient values and priorities. These concepts may be beneficial to all patients accessing ART services as empathetic care, efficiency, and improved clinical and counselling could prevent disengagement among those at risk. This may partly explain why the intervention was associated with a general improvement in clinic-level HIV outcomes compared to non-intervention clinics^3^.

Intervention awareness was inconsistent among HCWs and patients, which may indicate insufficient engagement in workshops, and the program not adequately shifting attitudes as intended. While clinical guidelines used in the *Welcome Service* remained unchanged from standard-of-care (instead, provided additional tools and training to support guideline uptake), implementation of existing guidance was mixed: half of eligible patients received TB prevention, cotrimoxazole prophylaxis, and CD4 testing at re-engagement. Despite low completion, cotrimoxazole prophylaxis was higher in this study (45%) than in another study in South Africa, where 23–26% of patients with a CD4 < 200 received it at ART initiation (another indication for prophylaxis)^44^. Nevertheless, same-day ART and switching to dolutegravir, factors known to improve VL suppression, were performed well and are arguably more important clinical practices to achieve treatment success than prophylaxes^45^. Guidelines use may vary due to many factors, including knowledge of and agreement with guidelines, ability to integrate recommendations into workflow, resource availability, and perception of patient needs^37,38,46^. TB and cotrimoxazole prophylactic therapies are frequently under-prescribed, evidenced in a study showing 63% TB prevention coverage among people newly initiated on ART in South Africa^28,47^. This may be due to provider-related barriers to prescription such as concerns about pill burden, treatment side effects and confidence in clinically excluding TB before committing to 6–12 months of prophylaxis^48,49^.

Counsellors with limited training provide most of the psychosocial support for PLHIV in low-resource settings^50^. *Welcome Service* counselling was highly valued by patients and HCWs, suggesting further investment and training are needed to fulfill this vital role. While no negative experiences of counselling were reported, treatment literacy gaps suggested patients may not have standardized access to counselling. Previous research finds adherence is associated with patients feeling their healthcare provider knows them well, further highlighting the importance of patient-provider relationships in engagement^51^, which can be undermined by the lack of continuity-of-care. The positive experience of *Welcome Service* counselling highlights further consultation is needed to restructure routine sessions typified by advice and information-sharing to patient-centered care.

Findings highlighted challenges in accurately measuring ART engagement, widely acknowledged in other literature^52,53^. Although VL is the gold standard measure for engagement, ranges are almost infinite, ‘suppression’ cut-offs are arbitrary and have varied vastly over the last two decades, making it difficult to compare between groups and with other studies^53^. A multi-study review showed moderate relation between VL and other engagement measures (such as retention and adherence) suggesting it may not be sensitive enough to truly reflect engagement^53^. Measuring engagement is complex and more comprehensive, standardized measures than the threshold of 1000c/mL (or 50c/mL) are needed^52^. Despite not being able to compare VL outcomes with a control, 88% of enrolled patients had a 4-month VL < 1000c/mL, close to the 95% UNAIDS target. This could be considered a successful public health intervention as the VL threshold for transmission, treatment resistance and advanced HIV is thought to be higher than the formerly used cutoff of < 50c/mL, particularly with the introduction of dolutegravir^52,54,55^.

In the *Welcome Service*, re-engaging patients had lower levels of retention (66%) than in other literature (73%) assessing outcomes following implementation of differentiated care models in sub-Saharan Africa^56^, which may be expected given the history of treatment interruption. This suggests people re-engaging with care should be prioritized for supportive interventions, as the biggest hurdle to *Eliminating HIV by 2023* may be to get people on ART rather than to maintain suppression among those in care^1^. Despite this, little literature explores the expected trajectory of patients after re-engagement, making target-setting challenging. At clinic-level, an overall improvement in retention and adherence was seen among the clinic population on ART, which could suggest the intervention had a broader impact beyond re-engaging patients. While the program was aimed at people re-engaging, aspects such as improved triage and welcoming and supportive staff had the potential to impact all patients accessing ART. However, the secondary analysis had limitations and should be interpreted with caution: comparisons were made among all patients on ART, not among those enrolled and did not use a control group.

There is increasing recognition that ART engagement is not linear, and disengagement is likely to affect most people on ART at some point in their lifetime^2^. While engagement is increasingly recognized as a priority for HIV policy and funding^57^, more urgent strategies should be deployed to prevent treatment interruption to realize the UNAIDS 2030 targets. The *Welcome Service* provides lessons for future programs to capitalize on current momentum. Concurrently, another NGO and South Africa’s DOH introduced the ‘Welcome Back Campaign’ that drew on lessons learned from the *Welcome Service* pilot^58^. It is evident that the *Welcome Service* intervention strategy and its execution did not fully address the challenge of disengagement in those returning to care, however, this evaluation demonstrates that some changes can be made at facility-level. It also highlights the complexity of patient-HCW relations, root challenges in the health system and broader social influences on engagement^2,3,15,59^. Further research is needed to identify the form that interventions to support re-engagement should take in different contexts, while adhering to the core function and principles around which the *Welcome Service* was designed.

This study contributes to our understanding of outcomes among patients re-engaging after ART interruption, a topic with limited research. The large sample size, use of quantitative and qualitative data, consideration of HCW and patient perspectives, and secondary evaluation of clinic-level outcomes, provides valuable insights for researchers and program designers when developing future interventions and setting targets for re-engaging patients. Qualitative data allowed us to unpack the impact of real and anticipated judgment and highlighted the challenge in making a meaningful shift toward empathic, person-centered care. Resource constraints and challenges introduced by the COVID-19 pandemic limited the ability to differentiate the effect of the four individual intervention components. With these challenges and the moderate achievement of implementation objectives, the true value of intervention could be underestimated. The pandemic may have also reduced the efficacy of the intervention as both patients and HCWs faced competing priorities and shifts in healthcare provision. Interviews were conducted shortly after patients’ re-engagement visit, missing an opportunity to explore longitudinal perspectives. Social desirability may have influenced responses among participants aware that MSF implemented the intervention^45^. Interviews were limited to re-engaging patients, missing the experiences of care among stable patients.

## Conclusion

This study showed that patients had largely positive experiences of care after the *Welcome Service*, was introduced although HCWs’ understanding of empathy and person-centered care did not always translate into their attitudes and behaviors. Without fully achieving the intervention’s overall goals, shown by the mixed achievement of implementation objectives, it was not possible to fully understand the potential impact the *Welcome Service* intervention. Nevertheless, with 88% of *Welcome Service* patients achieving VL suppression ≤ 1000 copies/mL at 4 months, and the positive clinic-level ART outcomes associated with the intervention, the *Service* may have had some impact on re-engaging patients and a wider impact on the general clinic population on ART. The core concept and principles of the *Welcome Service* merit exploration in an alternative program structure, based on the lessons from this implementation, to identify feasible, scalable services that support sustainable treatment success after re-engagement.

### Supplementary Information

Supplementary Information.

## Data Availability

Data management files can be made available upon request. Data used for this study will be stored in secure data repository by MSF for 5 years after the publication of findings. Due to the sensitivity of the data, data can only be made available upon request and approval by the Human Research Ethics Committees of the University of Cape Town and Médecins Sans Frontières Ethics Review Board.

## Abbreviations

AIDS: Acquired human immunodeficiency virus
ART: Antiretroviral therapy
CPT: Cotrimoxazole prevention therapy
DID: Difference in differences
DOH: Department of health
IQR: Interquartile range
HCW: Healthcare worker
HIV: Human immunodeficiency virus
MPR: Medication possession ratio
MSF: Médecins Sans Frontières
n: Number
PDC: People’s Development Center
PHDC: Provincial health data center
PLHIV: People living with HIV
TB: Tuberculosis
TPT: Tuberculosis prevention therapy
VL: Viral load
