# Supplementary material for: Supporting re-engagement with HIV services after treatment interruption in South Africa: a mixed method program evaluation of MSF’s Welcome Service
Source: Sci Rep. 2024 Mar 27;14:7317. doi: 10.1038/s41598-024-57774-9 (PMC10973441; doi:10.1038/s41598-024-57774-9)
Supplement: Supplementary file 1 — Supplementary Information. [file 41598_2024_57774_MOESM1_ESM.docx]

**Supplement 1.1: The Core Welcome Service Principles**


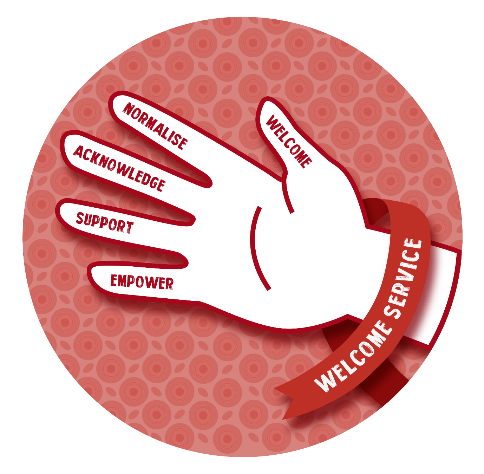
With negative staff attitudes being one of the predominant barriers to patients spontaneously re-engaging with care, the Welcome Service aimed to invest in capacitating and enabling staff to feel empowered to manage complex and potentially emotionally draining patients. A checklist was developed to shape the approach to these patients, i.e. the “Welcome Handshake”, consisting of five components:

1. Welcome the patient to the service and make them feel valued
2. Normalize the struggle with treatment and reduce their guilt at having disengaged
3. Acknowledge that they have returned to care, and build on this achievement
4. Support them in the ways that they need (individualized) to adhere to their treatment
5. Empower them to take ownership of their treatment

These components are emphasized in the healthcare worker engagement training and counsellor training. The health care worker engagement training is described further in an open access resource available at: <https://www.msf.org.za/news-and-resources/publications/msf-welcome-service-toolkit>

**Counselling**

Individual counselling sessions are provided by clinic counsellors, for whom the Welcome Service has developed a toolkit. Counsellors are equipped to provide 2 types of counselling sessions, including:

1. Return to Care Counselling

This occurs at the enrolment visit at re-engagement with care after interruption. The focus of this session is the 5 core principles demonstrated above: counsellors are encouraged to make the patient feel welcome, normalize their challenges with engagement and acknowledge what it has taken for them to come to the clinic today. Before diving into the reasons for disengagement and managing them, counsellors are taught to create a safe space for patients using these principles.

1. Treatment interruption counselling

Usually conducted at their second visit, counsellors are taught to focus on principles 4 and 5. They are guided to assess their patients’ individual needs, what lead them to disengage, determining a treatment plan to address their needs and support them to take ownership of their health in future, through empowerment and education.

**Supplement 1.2: Descriptions of data sources used.**

|  | **Process evaluation** | | **Outcome evaluation** |
| --- | --- | --- | --- |
| **Data source** | **Qualitative interviews** | **Program monitoring data** | **Routine Public Health Data** |
| **Data description** | In-depth interviews with healthcare workers trained on the *Welcome Service* or people re-engaging with ART who received the intervention (e.g., perceptions and experiences of the intervention) | Clinical monitoring data (i.e., names and folder numbers of people re-engaging with ART, restart regimen, CD4 count, prophylaxis.) | Data on patients accessing public health services in the Western Cape Province (e.g., healthcare visits, pharmacy dispensing, laboratory testing, imaging data etc.) |
| **Enrolment and study period** | Interviews conducted from December 2021 to May 2022 | 01/04/2021–30/06/2021 | 01/04/21–30/06/2021 (Study cohort)  01/04/19–30/06/2019 (Baseline cohort) |
| **Clinics included** | Healthcare workers and patients from the intervention clinics | Intervention clinics only | Intervention and control clinics |
| **Sampling and cohorts** | Patients were approached by a Médecins Sans Frontières nurse after receiving care and invited to be interviewed at a later date/time. Healthcare workers who received training were invited to participate by the MSF interviewer. | People who re-engaged with HIV care after a documented interruption (of >56 days late after an expected visit) and were enrolled in the *Welcome Service* intervention | All people who accessed any healthcare service for HIV (clinic visit, laboratory test or ART dispensed) during the study period at the intervention or control clinics, irrespective of having an interruption in care |
| **Anonymity** | Transcripts were anonymized before being analyzed. Patient identifiers were kept in a secure file accessible only two members of the research team. | Patients’ names and folder numbers were collected when creating a list of those enrolled. Identifiable data was only available to the epidemiologist. | All records were anonymized when provided to the Médecins Sans Frontières research team by the province. |
| **Evaluation** | Used to describe and evaluate knowledge, perceptions, attitudes, and implementation of the intervention. | Used to describe and evaluate the implementation of the intervention. | Used to evaluate the impact of the intervention on treatment outcomes (retention, adherence, and viral suppression). |
| *ART = Antiretroviral Therapy, HIV = Human immunodeficiency virus* | | | |

**Supplement 1.3: Calculation of 0–6-month medication possession ratio**

**Example 1:**

|  | Visit 1 | Visit 2 | Visit 3 | Visit 4 | Visit 5 | Visit 6 | Visit 7 | 6-months post enrolment |
| --- | --- | --- | --- | --- | --- | --- | --- | --- |
| Date of visit | 01-Apr-21 | 29-Apr-21 | 27-May-21 | 24-Jun-21 | 22-Jul-21 | 19-Aug-21 | 16-Sep-21 | 28-Sep-21 |
| Days after enrolment visit | 0 | 28 | 56 | 84 | 112 | 140 | 168 | 180 |
| Number of pills dispensed | 28 | 28 | 28 | 28 | 28 | 28 | 28 | NA |
| # of pills included in calculation | 28 | 28 | 28 | 28 | 28 | 28 | 12 | NA |

Total number of pills dispensed = 28 x 6 + 12 = 180 days

MPR = 180 days / 180 days = 100%

Note: The time between visit 7 and 6-months post enrolment is 12 days, therefore the maximum number of days of pills included in the calculation for that visit is capped

**Example 2:**

|  | Visit 1 | Visit 2 | Visit 3 | Visit 4 | Visit 5 | Visit 6 | Visit 7 | 6-months post enrolment |
| --- | --- | --- | --- | --- | --- | --- | --- | --- |
| Date of visit | 01-Apr-21 | 27-May-21 | 22-Jul-21 | 16-Sep-21 | No more visits | | | 28-Sep-21 |
| Days after enrolment visit | 0 | 56 | 112 | 168 |  |  |  | 180 |
| Number of pills dispensed | 56 | 28 | 28 | 28 |  |  |  | NA |
| Number of pills included in calculation | 56 | 28 | 28 | 12 |  |  |  | NA |

Total number of pills dispensed = 56 +28 +28 + 12 = 124 days

MPR = 124 days / 180 days = 68.9%

Note: The time between visit 4 and 6-months post enrolment is 12 days, therefore the maximum number of days of pills included in the calculation for that visit is capped

**Supplement 1.4: Characteristics of patients accessing ART services at the intervention and control clinics before and after the implementation.**

| Variable | | Intervention Clinics | | Control Clinics | |
| --- | --- | --- | --- | --- | --- |
|  |  | Before* (2019) | After* (2021) | Before (2019) | After (2021) |
| Total patients, n (%) | 31,718 | 7,291 | 7,623 | 9,798 | 7,006 |
| Female, n (%) | | 5,354 (73) | 5,564 (73) | 6,851 (70) | 4,931 (70) |
| Age in years (median) | | 41 (35-47) | 42 (36-48) | 40 (34-47) | 41 (35-48) |
| Last CD4 (cells/mm3) before study period, n (%) | CD4 available | 387 (5) | 2342 (31) | 1039 (11) | 3,003 (43) |
|  | <50 | 33 (9) | 120 (5) | 45 (4) | 92 (3) |
|  | 50-100 | 31 (8) | 127 (5) | 49 (5) | 120 (4) |
|  | 100-200 | 59 (15) | 275 (12) | 153 (15) | 296 (10) |
|  | 200-350 | 100 (26) | 591 (25) | 221 (21) | 621 (21) |
|  | >350 | 164 (42) | 1,229 (52) | 571 (55) | 1,874 (62) |
| ART regimen, n (%) | First-line with NNRTI | 6,547 (90) | 1,496 (20) | 8,328 (85) | 2,109 (30) |
|  | First-line with dolutegravir | 0 (0) | 5,325 (70) | 452 (5) | 4,070 (58) |
|  | Second line with a protease inhibitor | 699 (10) | 683 (9) | 889 (9) | 597 (9) |
|  | Second line with dolutegravir | 0 (0) | 90 (1) | 1 (<1) | 122 (2) |
|  | All other regimens | 42 (1) | 25 (<1) | 125 (1) | 99 (1) |
| *ART = antiretroviral therapy; n = Number; NNRTI = non-nucleoside reverse transcriptase inhibitors* **Before = patients accessing ART services between 01/04/2019-30/06/2019; After = patients accessing ART services between 01/04/2021-30/06/2021* | | | | | |

**Supplement 1.5: measuring parallel trends assumption.**

To evaluate the suitability of the difference in differences (DID) analysis, parallel trends were tested visually for all outcome variables using graphs in Stata 17.0. This includes retention (attendance), adherence (medication possession ratio) and viral suppression.

The baseline group (enrolled in 2019) was divided into three: those enrolled in April 2019, May 2019, and June 2019. Outcomes were tested by month to observe changes occurring from month to month.

2.1. Retention/clinic attendance

**Variables included as dependant/explanatory: age, sex

*Conclusion: there is sufficient certainty that the intervention and groups follow the same trend over time and therefore the parallel trend assumptions hold for retention (clinic attendance).*

2.2. Adherence/medication possession ratio (MPR)

Variables included as dependant/explanatory: age, sex

*Conclusion: there is sufficient certainty that intervention and control groups follow the same trend over time and therefore the parallel trend assumptions hold for MPR (adherence).*

2.3. Viral suppression

Variables included as dependant/explanatory: age, sex, regimen at restart

*Conclusion: there is insufficient certainty that the control and intervention groups follow the same trend over time and therefore the parallel trend assumption does not hold when analysing viral load or viral suppression as the outcome measure. The DID is therefore not a suitable analysis to test the null hypothesis.*

**
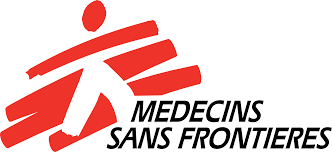
Supplement 2**


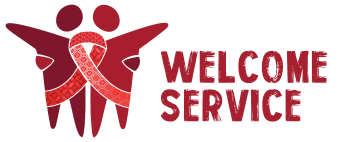

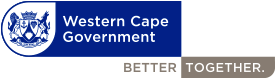


**The Welcome Service in Khayelitsha:**

**Supporting engagement with ART services**

**Overview:**

South Africa is home to the largest HIV cohort in the world, with 7.5 million people living with HIV, 5.3 million people on ART and 4.9 million who are virologically suppressed.^1^ Although access to ART services has dramatically changed over the last 20 years, 2.6 million people (36%) living with HIV in South Africa are still not accessing optimal ART services. Traditional WHO 90-90-90 targets make the assumption that patients are diagnosed with HIV, start ART and become virally suppressed. However, the reality is that patients cycle in and out of care throughout their treatment journey. Research shows that 25% of patients on ART in Khayelitsha will interrupt care at least once within a period of 2 years and 60% in a 10-year period.^2^ Many return to care on their own after interrupting treatment and health systems are not designed to adequately manage these patients when they return: they are more likely to have advanced HIV, treatment resistance and complicated psychosocial barriers to engagement. The Welcome Service is a differentiated service delivery model for HIV care, developed by MSF in collaboration with the Western Cape Department of Health, which aims to support engagement with ART services and long-term retention in care, to ultimately reduce HIV-related morbidity and mortality.

In a systematic review of qualitative literature, Eshun-Wilson *et al^3^* explores how different factors influence adherence and long term retention in HIV care. They describe how clients experience a multitude of competing stressors that combine to a point at which clients are unable to cope and “tip over” into disengagement (i.e. stop taking treatment or miss appointments). It also highlights the important role that healthcare workers play in ART engagement. Clients who have disengaged are at risk of advanced HIV, may be challenging to manage and time- or resource intensive: healthcare workers may feel overwhelmed by these clients and could lead to negative attitudes and authoritarian behaviours portrayed toward these patients. The Welcome Service, developed on these theories, aims to build patient resilience and promote long-term retention in ART services by creating a more patient-centered and non-judgmental approach to care and focusing on better identification and management of barriers to engagement. The package is designed to be integrated into routine health services, and is made up of 4 core components described below.

**Aim:**

Support effective engagement in ART services and long-term retention in care to ultimately reduced HIV-related morbidity and mortality.

**Objectives & Core Components:**

1. **Optimal Clinical Management of HIV in primary care**

Objectives:

- Support early and appropriate re-initiation on ART after interruption
- Support early and appropriate switching of ART regimens where necessary
- Screening and prevention of common opportunistic infections (TB, CM, cotrimoxazole)
- Promote primary care access to point of care testing for advanced HIV (TB Lam & LPs)

Tools for implementation

- Clinical staff
  - Clinical training package for NIMART-approved nurses or medical officers
  - One-on-one mentorship
- Non-clinical staff (e.g. admin, security guards, cleaners) – training package for non-clinical staff on basics of HIV, e.g.:
  - How to recognize a sick patient and what to do (i.e. appropriate/inappropriate triage)
  - Stigma
  - Confidentiality

1. **Improve identification, triage and monitoring of clients on ART**

Objectives:

- Reduce unnecessary delays
  - Prevent patients from waiting to be seen at the end of the day or being turned away
  - Make it easier for patients to navigate the complicated clinic system
  - Streamline patient care
- Improve triage system to identify clients needing support and ensuring they are streamlined into the service they need
- Improve monitoring & evaluation using electronic & automated data systems where possible

Tools for implementation

- Identification & Triage
  - Map patients’ journey through the clinic to identify unnecessary delays
  - Improve clinic flow to maximize efficiency, reduce delays and ensure patients go through the correct stream of care
- Monitoring & Evaluation
  - Training of admin staff, clerks, data capturers
  - Improve routine systems for monitoring and evaluation
  - Promote uptake of electronic and automatic data systems where possible

1. **Improve psychosocial support for clients on ART**

Objectives:

- Improve the identification and management of barriers to engagement
- Appropriately refer patients to mental health and social services where necessary (social work, substance use, gender based violence etc)
- Promote a more welcoming and patient centered approach to care (the handshake: WELCOME, NORMALIZE, ACKNOWLEDGE, SUPPORT, EMPOWER)

Tools for implementation

- Training package for counselors or clinicians with counseling responsibilities – adopted by the People’s Development Centre
- Counseling stationery tools – the “Treatment Interruption Tool”


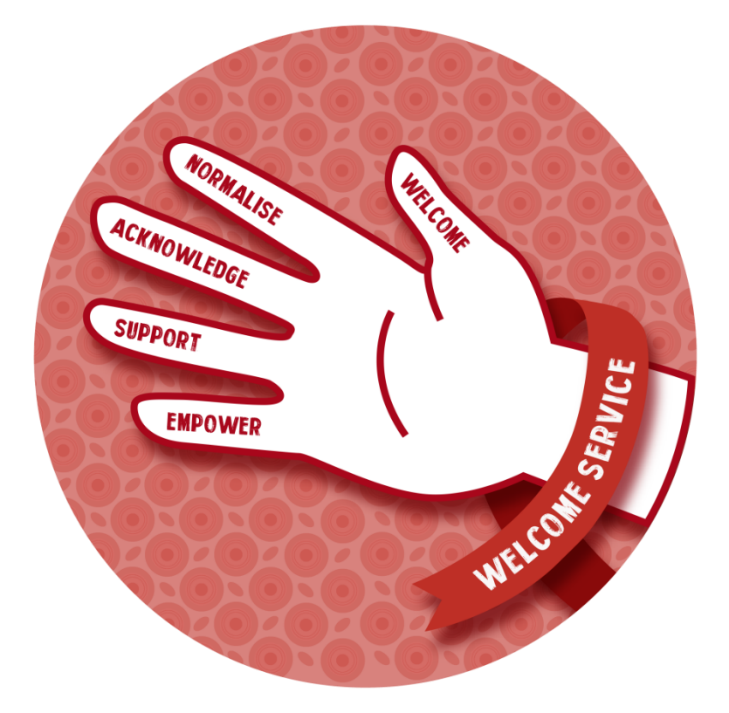


1. **Support healthcare worker engagement**

Objectives

- Engage on healthcare workers’ stressors & develop coping mechanisms
- Acknowledge and manage HCW’s challenges with managing these patients
- Recognize how feeling stressed and burned out can impact patient care
- Better empathize with patients’ difficulty in engagement and promote a more welcoming environment & non-judgmental attitude toward patients who struggle

Tools for implementation

- Training package – “Health Care Worker Engagement Package”

**Who is the Welcome Service for?**

Patients who have interrupted treatment, who may present as having:

- Missed appointments or medication collection
- Flagged as lost to follow up

**References**

1. Joint United Nations Programme against HIV & AIDS. *Global HIV & AIDS Statistics - Fact Sheet*. https://www.unaids.org/en/resources/fact-sheet (2021).
2. Kaplan, S. R. *et al.* Contemporary disengagement from antiretroviral therapy in Khayelitsha, South Africa: A cohort study. *PLoS Med.* **14**, 1–24 (2017).
3. Eshun-Wilson, I., Rohwer, A., Hendricks, L., Oliver, S. & Garner, P. Being HIV positive and staying on antiretroviral therapy in Africa: A qualitative systematic review and theoretical model. *PLoS ONE* **14**, 1–30 (2019).
